# Insecticide resistance modifies mosquito response to DEET and natural repellents

**DOI:** 10.1186/s13071-019-3343-9

**Published:** 2019-03-12

**Authors:** Emilie Deletre, Thibaud Martin, Claire Duménil, Fabrice Chandre

**Affiliations:** 1 Cirad - Montpellier University - UPR Hortsys, Montpellier, France; 2 International Centre of Insect Physiology and Ecology (icipe), Nairobi, Kenya; 3 UMR MIVEGEC, IRD-CNRS-Montpellier University, Montpellier, France

**Keywords:** *Anopheles gambiae*, Permethrin, Deterrent, Mortality, Geraniol, Cinnamaldehyde

## Abstract

**Background:**

Pyrethroid and organophosphate resistance in the malaria vector *Anopheles gambiae* has led to the search for not only alternative insecticides, but also repellent chemical compounds. However, little is known about the potential actions of repellents and the cross-resistance risk between insecticide and repellent compounds.

**Methods:**

Here we show the action of permethrin, DEET, geraniol, carvacrol, culminaldehyde and cinnamaldehyde against three *A. gambiae* strains: ‘Kis’ (Kisumu susceptible strain), ‘KdrKis’ (pyrethroid resistant strain) and ‘AcerKis’ (organophosphate resistant strain), the last two differing from the first by a mutation on the *kdr* and *ace1* genes, respectively.

**Conclusions:**

Results from the DEET assays show it induced repellency for the resistant KdrKis and AcerKis strains but maintained irritancy for the susceptible strain. More generally, we show resistance genes modify the behavior of *An. gambiae*, increasing or decreasing the effectiveness of DEET and natural compounds, depending on the mutation. These findings offer a new avenue for research on the target and mechanism of repellent compounds. We discuss these findings in the context of vector control strategies.

**Electronic supplementary material:**

The online version of this article (10.1186/s13071-019-3343-9) contains supplementary material, which is available to authorized users.

## Background

The *Anopheles gambiae* (Giles, 1902) complex includes major vectors responsible for the transmission of *Plasmodium* spp., causing malaria infections in humans [1]. The enormous progress in rapid diagnostic tests (associated with efficient treatments such as artemisinin-based combination therapy against *P. falciparum*) and vector control with indoor residual spraying or long-lasting insecticide treated nets, has resulted in an overall decrease in malaria deaths [2]. Relatively safe for humans, pyrethroids have rapid irritant (or excito-repellent), knockdown and killing effects [3], mediated by modifying the gating kinetic of the voltage-dependent sodium channel. Pyrethroids of type I (e.g. permethrin) prevent the sodium channels from closing, creating a succession of repetitive action potentials, while the sodium channels in type II pyrethroids (e.g. deltamethrin) stay open without producing repetitive discharge [4]. Pyrethroids can be used for indoor spraying, and they are also used for treating bednets or cloths [5]. A common form of resistance to pyrethroids in *An. gambiae* is knockdown resistance (*kdr*), resulting from a mutation (L1014F or L1014S) of the voltage-dependent sodium channel gene (Na_v_) [6, 7]. These mutations reduce the affinity of pyrethroids to Na_v_ [8]. Organophosphates (OPs) are mainly used for indoor residual spraying, although only a few are recommended by the World Health Organization. Indeed, OPs do not have any irritant or repellent effect, they kill the mosquitoes after landing on the wall surfaces. OP insecticides inhibit acetylcholinesterase, a key enzyme of the nervous system [9]. Acetylcholinesterase (AChE) degrades the neurotransmitter at the cholinergic nerve synapse. When inhibited, acetylcholine accumulates in the synaptic junction and the receptors remain open, inducing paralysis and death [10–12]. For several mosquito species (including *An. gambiae*), a G119S substitution in AChE1 encoded by *acwie-1* gene has been implicated in resistance to OP and carbamate insecticides [13–15]. The most common active ingredient in insect repellent (especially against mosquitoes), *N*,*N*-diethyl-*m*-toluamide (DEET), inhibits olfactory neuron receptors and masks attractive odours in *An. gambiae* [16, 17]. *In vitro*, DEET was also found to be an acetylcholinesterase inhibitor in mosquitoes [18], and Stanczyk et al. [19] also identified *Aedes aegypti* females that were insensitive to DEET, but no males, due to a genetically determined dominant trait and residing in changes in the sensillum function. Insecticide-based strategies have contributed to improving public health in many countries [20]. Nevertheless, vector control could be under the threat of continuous selection for resistant populations to insecticides. Indeed, pyrethroid and OP resistance have been reported in 27 countries from sub-Saharan Africa, with multiple resistance mechanisms, such as decreased sensitivity of the target protein and increased metabolic detoxification, underscoring the need to find alternatives to these chemical insecticides [2, 7, 9, 21–24]. Insecticide resistance can impact the behavioral response. Studies have previously shown that a *kdr*-resistant strain of *A. gambiae* is less affected by pyrethroids than the susceptible strain [25]. Although some studies showed that *kdr* resistance failed to decrease the effectiveness of insecticide-treated nets [26], others reported a fitness advantage for *kdr-*resistant phenotypes [22] that could decrease the efficacy of pyrethroid treated nets [27].

Generally, the search for novel compounds for vector control has focused on their toxic effects. Nevertheless, research on other effects (such as repellency or irritancy) that may be used to reduce vector-host contact are currently being undertaken, although few studies have focused on their effect on insecticide-resistant strains. In previous studies, we showed that (*E*)-cinnamaldehyde, the major component of cinnamon bark (*Cinnamomum zeylanicum*) essential oil; carvacrol, one of the major compounds of thyme leaf (*Thymus vulgaris*) essential oil; geraniol, one of the major compounds of citronella leaf (*Cymbopogon winterianus*) essential oil; and cuminaldehyde, the major compound of cumin seed (*Cuminum cyminum*) essential oil; could have a repellent, irritant and/or toxic effect on an *An. gambiae* susceptible strain (Kisumu) [28, 29]. The repellent chemicals identified to-date in plants are: (i) alkaloids that can affect the acetylcholine receptors in the nervous system [30] or membrane channels of nerves [31]; (ii) phenols, particularly in the flavonoid group; and (iii) terpenoids, the most important insect repellent group to consider. For example, monoterpenes penetrate the insect cuticle, which increases their bioavailability [32]. This property could be of interest if it resulted in shortened stay of insects on treated surfaces. Three pathways have been studied to explain the toxicity of essential oils: (i) the inhibition of the acetylcholinesterase; (ii) interference with the neuromodulator octopamine; and (iii) inhibition of GABA-gated chloride channels [33–35]. While the mode of action of repellency and irritancy has not been well studied, repellents could function through the activation (or inactivation) of olfactory receptor neurons and irritants through the activation of gustatory receptor neurons [36, 37].

The overall purpose of this article is to examine how insecticide resistance genes modify the behaviour of the mosquito *Anopheles gambiae* exposed to DEET and natural repellent compounds. Our specific aims are to identify the risk of cross-resistance between insecticides and repellents, and elicit more information about their potential mechanisms. In three behavioural assays, DEET and four bioactive repellent compounds were tested on a pyrethroid (*kdr* gene) and an OP (*ace1* gene) resistant strain compared to a susceptible one, in comparison with permethrin and non-treated control. In this study, we tested: (i) spatial repellency (also called expellent repellency) which corresponds to oriented movement of the insect away from a volatile chemical source without direct contact; (ii) contact repellency (also called irritancy landing inhibition or excito-repellency) which corresponds to oriented movement of the insect away from a chemical source with direct contact; and (iii) contact toxicity [37].

## Results

### DEET is a repellent to the *A. gambiae* resistant strain

DEET failed to show any repellency on the susceptible *Kis* strain at low and high doses but showed significant repellency at high concentration for the pyrethroid-resistant strains KdrKis (29.2%) and OP-resistant strain AcerKis (85.7%), compared with the non-treated control (Table 1, Additional file 1: Figure S1). Moreover, the repellent effect was significantly higher on the OP-resistant strain AcerKis than on the susceptible strain Kis (15%). DEET was an irritant at high concentration for all strains without significant difference between them (Table 2, Additional file 2: Figure S2). DEET was toxic at high concentration on the susceptible strain Kis (98.2%) and the OP-resistant strain AcerKis strain (96.6%), but not on the pyrethroid-resistant strain KdrKis (20.9%) (Table 3, Additional file 3: Figure S3).

**Table 1:** Repellent effect of DEET, permethrin, carvacrol, geraniol, cuminaldehyde and cinnamaldehyde on *Anopheles gambiae* from reference strains, the susceptible Kisumu strain (Kis), the pyrethroid-resistant strain (KdrKis) and the OP-resistant strain (AcerKis)

Product	Concentration (µl/cm^2^)	Kis strain^ab^	KdrKis strain^ab^	AcerKis strain^ab^
Permethrin	0.010	0.0 (0.0–4.9)	4.3 (0.0–10.6)	2.7 (0.0–9.6)
	0.100	6.3 (0.0–14.0)	11.0 (2.9–19.1)	8.0 (0.0–16.7)
DEET	0.010	0.0 (0.0–1.4)	20.1 (9.2–31.0)	11.9 (2.6–21.2)
	0.100	15.0 (5.2–24.7)	**29.2 (17.4–40.9)**	85.7 (77.6–93.8)*
Carvacrol	0.001	9.4 (1.7–17.0)	11.2 (3.1–19.3)	5.7 (0.0–13.5)
	0.014	**36.6 (25.0–48.2)**	**42.0 (30.1–53.8)**	0.0 (0.0–3.6)*
Geraniol	0.002	5.1 (0.0–13.0)	3.1 (0.0–9.5)	**22.1 (9.4–34.8)**
	0.023	**50.5 (38.0–63.1)**	**41.0 (28.9–53.1)**	9.7 (0.0–20.2)*
Cuminaldehyde	0.003	1.6 (0.0–4.7)	24.7 (12.6–36.8)*	8.5 (0.6–16.5)
	0.030	**25.4 (14.3–36.5)**	52.9 (41.0–64.9)*	**47.0 (35.2 - 58.8)**
Cinnamaldehyde	0.008	16.5 (7.8–25.3)	**36.2 (24.0–48.4)**	**37.7 (24.9–50.5)**
	0.079	**43.0 (32.1–54.0)**	82.1 (74.2–90.0)*	**53.1 (40.9–65.3)**

^a^Proportion of escaping mosquitoes as a percentage corrected with the negative control to compare strains by pairs (confidence interval)

^b^Pairwise comparison of proportion was done using Fisher’s test with the Holm’s sequential Bonferroni correction method. Values in bold lettering are significantly different from the non-treated control. Values followed by an asterisk (*) are significantly different from the Kis strain

**Table 2:** Irritant effect of DEET, permethrin, carvacrol, geraniol, cuminaldehyde and cinnamaldehyde on *Anopheles gambiae* from reference strains, the susceptible Kisumu strain (Kis), the pyrethroid-resistant strain KdrKis and the OP-resistant strain AcerKis

Product	Concentration (µl/cm^2^)	Kis strain^ab^	KdrKis strain^ab^	AcerKis strain^ab^
Permethrin	0.010	**37.7 (25.0–50.4)**	3.0 (0.0–7.9)*	10.1 (0.0–21.0) *
	0.100	**25.9 (13.8–38.0)**	**45.6 (33.7–57.4)**	**27.9 (16.7–39.1)**
DEET	0.010	7.6 (0.0–15.5)	22.6 (11.3–33.8)	16.5 (7.3–25.7)
	0.100	**54.1 (42.6–65.7)**	**71.2 (62.1–80.3)**	**43.6 (32.1–55.2)**
Carvacrol	0.001	**50.0 (38.2–61.8)**	0.1 (0.0–7.7)*	13.9 (5.2–22.7)*
	0.014	**84.3 (77.4–91.2)**	45.3 (33.4–57.2)*	49.3 (37.2–61.5)*
Geraniol	0.002	16.5 (5.2–27.7)	24.8 (14.5–35.2)	19.6 (9.1–30.2)
	0.023	**45.9 (41.4–50.4)**	73.9 (65.7–82.0)*	**41.8 (30.4–53.3)**
Cuminaldehyde	0.003	10.4 (1.1–19.6)	20.2 (9.5–31.0)	9.6 (1.9–17.2)
	0.030	**77.5 (69.4–85.6)**	**63.0 (53.0–73.0)**	46.3 (35.2–57.5)*
Cinnamaldehyde	0.008	22.0 (12.3–31.7)	23.8 (12.7–34.9)	11.7 (1.4–21.9)
	0.079	**63.2 (52.9–73.4)**	**70.4 (60.6–80.3)**	**51.1 (38.4–63.8)**

^a^Proportion of escaping mosquitoes in percentage corrected with the negative control to compare strains by pairs (confidence interval)

^b^Pairwise comparison of proportion was done using Fisher’s test with the Holm’s sequential Bonferroni correction method. Values in bold lettering are significantly different from the non-treated control. Values followed by an asterisk (*) are significantly different from the Kis strain

**Table 3:** Toxic effects of DEET, permethrin, carvacrol, geraniol, cuminaldehyde and cinnamaldehyde on *Anopheles gambiae* from reference strains, the susceptible Kisumu strain (Kis), the pyrethroid-resistant strain KdrKis and the OP-resistant strain AcerKis

Product	Concentration (µl/cm^2^)	Kis strain^ab^	KdrKis strain^ab^	AcerKis strain^ab^
Permethrin	0.010	**93.8 (89.7–97.8)**	2.7 (0.0–9.6)*	66.1 (53.7–78.5)*
	0.100	**96.8 (96.8–96.8)**	63.2 (49.8–76.5)*	**100.0 (100.0–100.0)**
DEET	0.010	2.6 (0.0–8.6)	0.0 (0.0–1.2)	0.0 (0.0–0.0)
	0.100	**98.2 (98.2–98.2)**	20.9 (9.7–32.0)*	**96.6 (96.6–96.6)**
Carvacrol	0.001	4.3 (0.0–9.0)	3.3 (0.0–9.8)	0.0 (0.0–0.0)
	0.014	0.0 (0.0–0.0)	0.0 (0.0–0.0)	0.0 (0.0–0.0)
Geraniol	0.002	0.0 (0.0–3.6)	1.7 (0.0–4.9)	0.0 (0.0–4.0)
	0.023	0.0 (0.0–3.5)	3.1 (0.0–7.3)	1.2 (0.0–9.3)
Cuminaldehyde	0.003	0.0 (0.0–0.0)	1.3 (0.0–5.5)	0.0 (0.0–0.0)
	0.030	11.1 (3.4–18.9)	3.8 (0.0–9.9)	30.9 (17.4–44.5)*
Cinnamaldehyde	0.008	0.0 (0.0–0.0)	0.0 (0.0–0.0)	0.2 (0.0–3.8)
	0.079	**45.9 (32.9–58.8)**	93.9 (87.2–100.6)*	89.9 (81.9–97.9)*

^a^Proportion of dead mosquitoes in percentage corrected with the negative control to compare strains by pairs

^b^Pairwise comparison of proportion was done using Fisher’s test with the Holm’s sequential Bonferroni correction method. Values in bold lettering are significantly different from the non-treated control. Values followed by an asterisk (*) are significantly different from the Kis strain

### Insecticide resistance modifies mosquito behavior to natural compounds

Carvacrol showed a significant repellent effect at high concentration for the susceptible strain Kis and the pyrethroid-resistant KdrKis strain, but not on the OP-resistant AcerKis strain (Table 1, Additional file 1: Figure S1). Carvacrol was an irritant at high concentration on the three strains but significantly less for both resistant strains KdrKis (45.3%) and AcerKis (49.3%), when compared to the susceptible strain Kis (84.3%) (Table 2, Additional file 2: Figure S2). Actually, carvacrol was an irritant at low concentration on the susceptible strain Kis but not on both resistant strains. Carvacrol was not toxic for any strains at low and high doses (Table 3, Additional file 3: Figure S3).

Geraniol had a significant repellent effect at high concentration for the susceptible strain Kis and the pyrethroid-resistant KdrKis strain, but not at low concentrations (Table 1, Additional file 1: Figure S1). Surprisingly, it was the opposite for the OP-resistant AcerKis strain, as geraniol showed repellent activity at the lower dose but not at the higher dose. Geraniol showed a significant irritant effect at high concentration on the three strains (Table 2, Additional file 2: Figure S2). Moreover, geraniol showed significantly more irritant effect on the pyrethroid-resistant strain KisKdr (73.9%) than on the susceptible strain Kis (45.9%). Geraniol was not toxic for any strains at both doses (Table 3, Additional file 3: Figure S3).

Cuminaldehyde showed significantly more repellent activity for all strains at high concentration (Table 1, Additional file 1: Figure S1). Whatever the concentration (low or high), cuminaldehyde was significantly more repellent on the pyrethroid-resistant strain KdrKis (24.7 and 52.9%, respectively) than on the susceptible strain Kis (1.6 and 25.4%, respectively). Cuminaldehyde exhibited an irritant effect at high concentration on the three strains (Table 2, Additional file 2: Figure S2) but was significantly less of an irritant on the OP-resistant strain AcerKis (46.3%) than on the susceptible strain Kis (77.5%). Cuminaldehyde was not toxic for any strains at both doses, compared with the non-treated control (Table 3, Additional file 3: Figure S3). However, at a higher dose, cuminaldehyde was significantly more toxic on the OP-resistant strain AcerKis (30.9%) than on the susceptible strain Kis (11.1%).

Cinnamaldehyde showed significantly more repellent activity for all strains at high concentration (Table 1, Additional file 1: Figure S1). The compound showed significantly more repellency on the pyrethroid-resistant strain KdrKis (82.1%) than on the susceptible strain Kis (43.0%). At low concentration, cinnamaldehyde was still repellent on both resistant strains KdrKis and AcerKis, but not on the susceptible Kis strain, when compared with the non-treated control. Cinnamaldehyde showed an irritant effect at high concentration on the three strains, but not at the lower dose (Table 2, Additional file 2: Figure S2). At the higher dose, cinnamaldehyde was toxic for all the strains as compared with the non-treated control (Table 3, Additional file 3: Figure S3), but cinnamaldehyde was significantly more toxic on the pyrethroid-resistant strain KdrKis (93.9%) and the OP-resistant strain AcerKis (89.9%) than on the susceptible strain Kis (45.9%).

### Permethrin is an irritant and toxicant rather than a repellent

At either dose, permethrin (control) did not act as a repellent for any strain, whether susceptible or resistant, compared with the non-treated control (Table 1, Additional file 1: Figure S1). At low concentration, permethrin showed a significant irritant action on the susceptible Kis strain (37.7%) but not on the pyrethroid-resistant strain KdrKis (3.0%) and OP-resistant strain AcerKis (10.1%). However, permethrin at a high concentration had an irritant effect for every strain compared with the non-treated control (Table 2, Additional file 2: Figure S2). As expected, permethrin at low concentration was significantly toxic to Kis and AcerKis but not to KdrKis compared with the non-treated control (Table 3, Additional file 3: Figure S3). At high concentration, permethrin was significantly toxic for all strains but significantly less for the pyrethroid-resistant strain KdrKis (63.2%) than for the susceptible Kis strain (96.8%) (Table 3, Additional file 3: Figure S3).

## Discussion

DEET elicited no spatial repellent action for the susceptible strain Kis. Our results show, for the first time, a spatial repellent effect of DEET for the pyrethroid-resistant strain KdrKis and a significantly greater effect for the OP-resistant strain AcerKis. DEET was an irritant for the resistant strains, suggesting a lack of cross-resistance. We observed a reduction of toxicity on KdrKis, but DEET is generally not used for that purpose. Although the mode of action of DEET has not been fully elucidated, it is known that this chemical interacts with several targets from the nervous system. Moreover, repellents can be defined in two different ways: a compound that causes a movement away from the odor source (spatial repellent) (no host odor in the bioassay) but also by a compound that prevents the recognition/location of the host (masking repellent), DEET could be a repellent of the second category [37]. The different kind of repellent and their associated bioassays are discussed in more details in [37]. DEET is a famous repellent but to our knowledge there are no publications describing a method to test DEET without a host (human, guinea pig, etc.); it has only been tested with a host, e.g. in the arm assay, hence considering only the masking effect and not the spatial effect. Actually, DEET is also known to inhibit olfactory neuron receptors, masking attractive odours in *An. gambiae* [16, 17]. However, a recent study showed that DEET activates the *Ir40a*^*+*^ neurons in *Drosophila melanogaster* and when *Ir40a* receptors are silenced, flies lose the ability to avoid DEET [38]. Within the central nervous system DEET targets octopaminergic synapses and affects muscarinic receptors [39, 40]. DEET was identified as an acetylcholinesterase inhibitor when tested on neurons *in vitro* [18] but from our study, there was no evidence that the repellent effect of DEET interferes with acetylcholinesterase activity *in vivo*. Our results suggest that acetylcholinesterase mutation (G119S) could enhance the DEET activity and increase its repellency against AcerKis. The similar high toxicity of DEET observed on AcerKis and Kis also suggests either that the steric effect of G119S mutation on acetylcholinesterase does not interfere with DEET affinity or that the primary target for DEET toxicity is not acetylcholinesterase, but other receptors from the central nervous system (e.g. octopamine receptors) [39]. Surprisingly, a significant reduction of DEET toxicity was observed against KdrKis sharing the same genetic background with Kis but being homozygous for L1014F mutation in the Na_v_ channels. This suggests that DEET has more than a single mode of action and that complex interactions between different targets from the nervous system are involved in its toxic effect and would require further neurophysiologic investigations.

Our results show that insecticide resistance alleles (such as *kdr* and *ace1*) could have a positive or negative impact on the effectiveness of carvacrol, geraniol, cuminaldehyde and cinnamaldehyde on the mosquito *Anopheles gambiae*, by modifying its behaviour (Table 4). We investigated the effects of these four natural bioactive compounds from essential oils, because of their promising effects on *Kis*, the susceptible strain of *An. gambiae* [29]. We observed a potentiation of the following effects on the pyrethroid-resistant strain KdrKis, compared with the susceptible strain: (i) an increase of the repellent effect of cuminaldehyde and cinnamaldehyde; (ii) an increase of the geraniol irritant effect, but a reduction of carvacrol effect; and (iii) an increase of cinnamaldehyde toxicity. On the OP-resistant strain AcerKis, we observed a reduction of the following compound effects: (i) a decrease of repellent effect for carvacrol, geraniol and cinnamaldehyde; (ii) a decrease of carvacrol and cuminaldehyde irritancy; and (iii) an increase of cinnamaldehyde toxicity. Comparison between the four bioactive compounds on a susceptible strain and two resistant strains showed differential effects that could be associated with the resistance mechanisms involved. Nowadays, few studies are conducted on the effect of natural compounds on the nervous system of insects. Most studies have investigated the effects of geraniol or carvacrol on AChE. Lopez & Pascual-Villalobos [41] showed that geraniol was a weak reversible competitive inhibitor of AChE, although it has a strong insecticidal property on *Sitophilus oryzae*, *Rhyzopertha dominica* and *Cryptolestes pusillus*. Other studies deal with the inhibition of TRP channels that are involved in neurophysiological processes (such as photoreception, pheromone sensing, gustative perception, thermosensation, pain perception and mechanosensation [42]). Cinnamaldehyde and carvacrol inhibit some TRP channels in *Drosophila* sp. [43, 44], suggesting that these natural compounds could act on several targets in the nervous central system. Geraniol had a lower repellent effect on *acerKis*, suggesting a possible interaction with the mutation of AChE. Cuminaldehyde, which is also an inhibitor of AChE [45], had a lower irritant effect and a higher toxic effect on AcerKis. The reduction in irritant effect tends to increase the contact of mosquitoes with the treated surfaces, and could contribute to increased mortality. In our study, carvacrol repellency and irritancy were weaker for the resistant AcerKis than the susceptible Kis. *In vitro*, carvacrol caused a slight inhibition of acetylcholinesterase from electric eels [46], and some arthropods (such as houseflies, ticks and cockroaches), but not from the mosquito *Aedes aegypti* [47]. The repellent effect of cinnamaldehyde and cuminaldehyde was higher on the strain with *kdr* mutation than on the two others. These two compounds induced a strong response using an electroantennogram (EAG), suggesting a possible interaction with specific odor receptors and/or Na_v_ channels [29]. In a previous study, we showed that geraniol elicited a significant response compared to ethanol using EAG, thus differing from carvacrol [29]. Since geraniol was also more of an irritant for KdrKis than for the other two strains, the mutated Na_v_ channel could potentiate the interaction with geraniol, and afterward its irritancy. Conversely, the carvacrol irritant effect was lower for the KdrKis resistant strain. Because these compounds act differently on susceptible and resistant strains, this suggests that CvpdNa and AChE could be primary or secondary targets of these compounds. Target site mutations could modify the sensitivity to these compounds, i.e. pleiotropic effects associated with resistance mechanisms that modify the behavioral response of resistant insects compared to susceptible ones. More studies on the affinity of natural compounds with the different receptors of the nervous system could enable researchers to identify new targets for repellent compounds or facilitate the discovery of new active molecules. The mode of action of these compounds should be further studied to determine how they first enter the insect, whether through ingestion, the respiratory route or cuticle absorption; for instance, volatile and/or topical routes of products can cause toxicity [48]. Some essential terpenes are also competitive inhibitors of acetylcholinesterase *in vitro*, but that may not correlate with toxicity, as evidenced by the case of carvacrol [33].

**Table 4:** Summary of repellent, irritant and toxic effects of permethrin, DEET, carvacrol, geraniol, cuminaldehyde and cinnamaldehyde on *Anopheles gambiae* from reference strains, the susceptible Kisumu strain (Kis), the pyrethroid-resistant strain KdrKis and the OP-resistant strain AcerKis

Product	Susceptible strain *Kis*	Pyrethroid-resistant strain *kdrKis*	OP-resistant strain *acerKis*
Permethrin	R^0^ I^+^ T^++^	↓ R^0^ I^−^ T^−^	↓ R^0^ I^−^ T^+^
DEET	R^0^ I^+^ T^+^	↑ R^+^ I^+^ T^0^	↑ R^++^ I^+^ T^+^
Carvacrol	R^+^ I^++^ T^0^	↓ R^+^ I^−^ T^0^	↓ R^0^ I^−^ T^0^
Geraniol	R^+^ I^+^ T^0^	↑ R^+^ I^++^ T^0^	↓ R^−^ I^+^ T^0^
Cuminaldehyde	R^+^ I^++^ T^0^	↑ R^++^ I^++^ T^0^	↓ R^+^ I^+^ T^0^
Cinnamaldehyde	R^+^ I^+^ T^+^	↑ R^++^ I^+^ T^++^	↑ R^+^ I^+^ T^++^

*Abbreviations*: R, repellent; I, irritant; T, toxic; 0, no effect; +, effect; ++, higher effect; −, lower effect; ↓, reduced efficiency for the strain; ↑, greater efficiency for the strain compared with the susceptible strain Kis

Our results confirm that permethrin is irritant and toxic but does not have a repellent effect for *Anopheles gambiae* [28, 29] as it does for other mosquito species (such as *An. albimanus* [49] or *Aedes aegypti* [50]). Indeed, permethrin is unlikely to volatilize based on its low vapor pressure (6.9 × 10^−6^ Pa at 25 °C) and low Henry’s law constant [51]. Our results show permethrin at the lowest dose is less irritant and toxic for the pyrethroid-resistant strain KdrKis than for the susceptible strain Kis. This was also observed for the OP-resistant AcerKis strain, but at a lower intensity than for KdrKis, taking into account the knocked-down individuals. At a higher dose, permethrin was less of an irritant for the Kis strain, because the knockdown effect of some mosquitoes (~30% individuals) during exposure prevented them from escaping, whereas permethrin became an irritant for the resistant KdrKis and AcerKis strains that failed to suffer knockdown. No knockdown effect was observed for the repellents. The knockdown effect is an early response of insects to an insecticide, leading to incapacitation, and occasionally, metabolic recovery [52]. Our results on the irritant effect of permethrin were influenced by its high and rapid toxicity, i.e. Kis females could not escape the cylinders, since a significant proportion were knocked down, whereas KdrKis were not knocked down and could escape. As previously reported by Chandre et al. [25], we confirmed that *A. gambiae* having *kdr* mutation is resistant to the toxic and irritant effects of pyrethroid compared to the susceptible strain. Our results show, to a lesser extent, quite similar effects on the OP-resistant strain AcerKis with the lowest dose of permethrin. While the reduction of toxic and irritant effects on the KdrKis-resistant strain threatens the effectiveness of insecticide nets treated with pyrethroids, our results and observations from the field suggest that when the dose is high enough, the reduced toxic effect is counter-balanced by the reduced irritancy, which increases the contact of mosquitoes with treated nets, leading to significant mortality of *kdr*-resistant mosquitoes. Hence, we showed that for type I pyrethroids (e.g. permethrin) the behavioural response of the KdrKis*-*resistant mosquito strain is modified, thus we can hypothesize that the behavioural response of the KdrKis*-*resistant mosquito strain will also be different toward type II pyrethroids (e.g. deltamethrin). Actually these pyrethroids have the same target, the sodium channels [4].

## Conclusions

We show for the first time a repellent effect of DEET on the pyrethroid-resistant strain KdrKis of the mosquito *Anopheles gambiae*, and more significantly for the OP-resistant strain AcerKis, compared to the susceptible strain Kis. Insecticide resistance genes (such as *kdr* and *ace1*) could have a positive or negative impact on the effectiveness of natural repellent compounds such as carvacrol, geraniol, cuminaldehyde and cinnamaldehyde on *An. gambiae*, by modifying its behaviour. Although the mechanisms underlying the role of target site mutations on the response to irritant or repellent compounds are not yet known, it has already been described in the case of pyrethroid resistance. Wagman et al. [53] showed that *Aedes aegypti* with a decreased insecticide susceptibility were insensitive to the repellent effect of transfluthrin. In the same way, pyrethroid-resistant strains of *An. gambiae* or *Culex quinquefasciatus* were less irritated by permethrin compared to susceptible ones [22, 25–54]. This study underlines the importance of testing new compounds on strains with known resistance mechanisms, even if they do not seem to have strong effects on the strains, since the behavioural response of insects to repellent or irritant compounds can be higher or lower, depending on their target and mode of action. Our evidence highlights the need for improved knowledge on the modes of action of repellent/irritant products that could be promising alternatives or complementary tools, to overcome insecticide resistance in mosquito populations.

## Methods

### Insects

Behavioural assays were performed using females of three reference strains of *An. gambiae*. The susceptible reference strain Kis (Kisumu susceptible strain) originally collected in Kisumu, Kenya in 1953, has been reared at LIN-IRD, Montpellier, France for more than 15 years. The insecticide susceptibility of the Kis strain was confirmed with World Health Organization (WHO) diagnostic doses (i.e. 4% DDT, 0.75% permethrin) and is regularly controlled every 4 months as recommended by ISO 9001. The colony was maintained in a climate-controlled room at 27 ± 2 °C, 80 ± 10% RH, with a photoperiod cycle of 12 h light: 12 h dark. Mosquito larvae were fed a diet of fish food (TetraMin, Tetra, Montpellier, France). Emerged adults were mechanically aspirated and transferred into 25 × 25 × 25 cm cages and provided access to 10% honey-water solution. The biological assays were also performed on females of *An. gambiae* from two resistant strains: the pyrethroid-resistant strain KdrKis (which is homozygous for the *kdr* L1014F mutation with the same genetic background as the Kis strain) and the organophosphate/carbamate-resistant strain AcerKis (which is homozygous for G119S *ace-1* mutation and has the same genetic background as the susceptible Kis strain) [5, 6]. The susceptible and resistant populations were reared in separate insectaries.

### Products

Studies were performed with four natural products: (*E*)-cinnamaldehyde (99% purity), geraniol (98% purity), cuminaldehyde (98% purity) and carvacrol (≥ 98% purity) (Sigma-Aldrich, St Louis, MO, USA). Two synthetic products were also tested: *N*,*N-*diethyl-3-methylbenzamide (also known as diethyltoluamide) (DEET, 97% purity) and permethrin (99% purity) from Sigma-Aldrich. The pyrethroid permethrin (mainly used in mosquito nets) and the insect repellent DEET (which is effective at reducing mosquito bites [55–57]), were used as positive controls. DEET and permethrin were diluted at 0.1 and 1% (v/v) in a solvent that consisted of ethanol (2/3) and Dow Corning® 556 cosmetic grade fluid (1/3). In a previous study we proved the four natural compounds (cinnamaldehyde, culminaldehyde, geraniol and carvacrol) were repellent, irritant and/or toxic at the relative concentration of 0.1% found in their respective essential oils (cinnamon, cumin, lemongrass and thyme essential oils [28, 29]). These products were tested at 2 concentrations: this efficient concentration, and diluted 10 times. These dilutions ensured that the quantity of a compound tested was approximately the same within the essential oil. Cinnamaldehyde was tested at 0.008 µl/cm^2^ of chromatograph paper and 0.079 µl/cm^2^, cuminaldehyde at 0.003 and 0.030 µl/cm^2^, geraniol at 0.002 and 0.023 µl/cm^2^, and carvacrol at 0.001 and 0.014 µl/cm^2^. Evaluation of a negative control with the solvent ethanol-silicone fluid preceded each assay (Table 2, Additional file 2: Figure S2). In repellency assays, 3.3 ml of this solution was deposited on a 13 × 30 cm chromatography paper, except on a border margin of 1.5 cm width. For irritancy and toxicity assays, 2 ml of the solution was deposited on 12 × 15 cm chromatography paper.

### Behavioral bioassays

Detailed descriptions of the apparatus, assay protocols, and data analysis procedures have already been published [28], modified from Grieco et al. [58]. In summary, bioassays were conducted between 10:00 and 18:00 h at 24 ± 1 °C and 50 ± 10% RH, and for each product; all assays were performed the same day.

### Repellency assays

The apparatus was a cylinder divided into two chambers, one treated and the other untreated. Treated papers (with products or with only the solvent, as control) were rolled around to cover the inner surface of the treated chamber, whereas untreated chromatograph paper was used to cover the inner surface of the untreated chamber. A metallic grid prevented direct mosquito contact with the treated paper. Twenty non-blood-fed females (aged 4–7 days-old) were introduced in the treated chamber and after a 30 s acclimation period, the butterfly valve separating the two chambers was opened for 10 min. At the end of the test, the butterfly valve was closed and the number of insects in each chamber recorded. Mosquitoes moving from the treated chamber to the untreated chamber were recorded as ‘escaped’. Conversely, mosquitoes remaining in the treated chamber were recorded as ‘stayed’. Tests were replicated three times for each chemical.

### Irritancy assays

These assays were performed using the system described for the repellent assay, and consisted of two connected tubes used in the WHO test kit, and a possible mosquito contact with the chemical. Ten non-blood-fed females (aged 4–7 days-old) were introduced in the treated chamber and each test performed six times for each chemical. After a 30 s acclimation period, the guillotine valve separating the two chambers was opened for 10 min, allowing the mosquitoes to move freely throughout the arena. Once the guillotine valve was closed, the number of mosquitoes in each tube (‘stayed’ *vs* ‘escaped’) was recorded.

### Toxicity assays

Toxicity assays were performed using a WHO Test Kit [59]. Twenty non-blood-fed females (aged 4–7 days-old) were exposed for 1 h to a treated paper (with products or with the solvent only) in the treated tube. Mosquitoes were then transferred to an untreated tube with 10% honey solution and maintained at 27 °C and 80% RH. The number of dead and alive *An. gambiae* was recorded after 24 h. Each test was replicated three times for each chemical.

### Statistical analysis

The same method was used to analyse the proportion of dead mosquitoes in toxicity assays and the proportion of escaped mosquitoes in both repellency and irritancy assays. Data analysis was carried out using R software v.2.12.2. Tests of treatment effects for the different behavioural assays were carried out on the proportion of escaped or dead mosquitoes in (i) control and treated assays; and (ii) susceptible and resistant strain assays. Fisher’s exact test with Bonferroni correction using Holm’s sequential method [60] was used for repellency and irritancy. The behavioural and mortality data were corrected using Sun-Shepard’s formula before comparing the susceptible strain with the resistant ones [61].

## Additional files

**Additional file 1: Figure S1.** Repellent effect of DEET, permethrin, carvacrol, geraniol, cuminaldehyde and cinnamaldehyde on *Anopheles gambiae* from the susceptible Kisumu strain (*Kis*), the pyrethroid resistant strain KdrKis and the OP resistant strain AcerKis.

**Additional file 2: Figure S2.** Irritancy effect of DEET, permethrin, carvacrol, geraniol, cuminaldehyde and cinnamaldehyde on *Anopheles gambiae* from the susceptible Kisumu strain (Kis), the pyrethroid resistant strain KdrKis and the OP resistant strain AcerKis.

**Additional file 3: Figure S3.** Toxicity effect of DEET, permethrin, carvacrol, geraniol, cuminaldehyde and cinnamaldehyde on *Anopheles gambiae* from the susceptible Kisumu strain (Kis), the pyrethroid resistant strain KdrKis and the OP resistant strain AcerKis.

## Abbreviations

Na_v_: voltage-dependent sodium channel gene
Kis: Kisumu susceptible strain
KdrKis: pyrethroid resistant strain
AcerKis: organophosphate resistant strain
OPs: organophosphates
AChE: acetylcholinesterase
WHO: World Health Organisation
DEET: *N*,*N-*diethyl-3-methylbenzamide
RH: relative humidity
EAG: electroantennogram
