# Supplementary material for: Insecticide resistance modifies mosquito response to DEET and natural repellents
Source: Parasit Vectors. 2019 Mar 12;12:89. doi: 10.1186/s13071-019-3343-9 (PMC6417241; doi:10.1186/s13071-019-3343-9)
Supplement: Supplementary file 2 — Additional file 2: Figure S2. Irritancy effect of DEET, permethrin, carvacrol, geraniol, cuminaldehyde and cinnamaldehyde on Anopheles gambiae from the susceptible Kisumu strain (Kis), the pyrethroid resistant strain KdrKis and the OP resistant strain AcerKis. [file 13071_2019_3343_MOESM2_ESM.pdf]

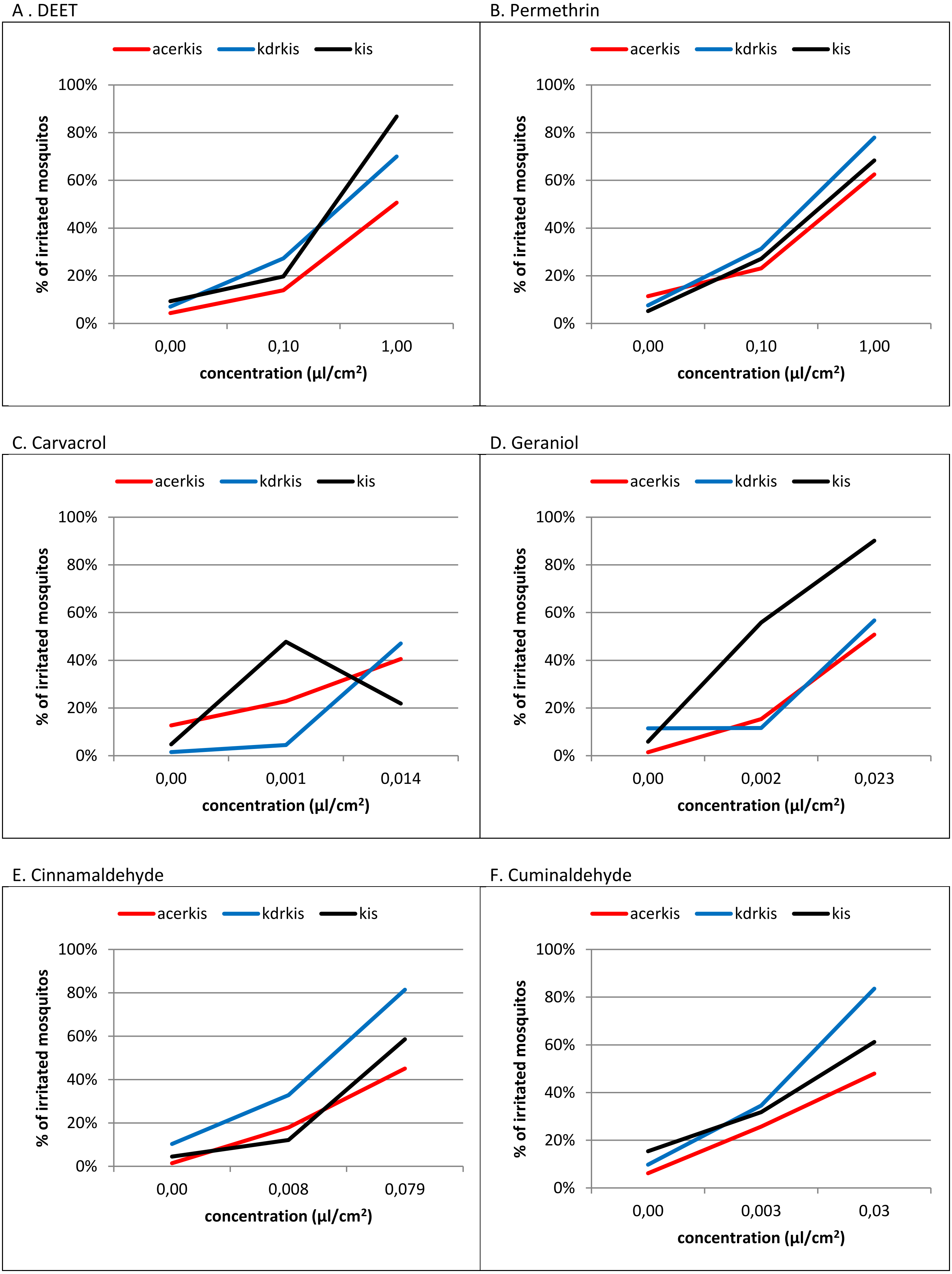

Additional file 2 : Irritant effect of DEET, permethrin, carvacrol, geraniol, cuminaldehyde and cinnamaldehyde *Anopheles gambiae*[1] from reference strains[2]

1) 4–7-day-old, non-blood-fed, sugar-fed, female mosquitos

2) The susceptible Kisumu (*Kis*), the pyrethroid resistant strain *kdrKis* and the OP resistant strain *acerKis*.
